# Early replication fragile sites are associated with cancer-related CNVs and SNVs in human embryonic stem cells

**DOI:** 10.1016/j.stemcr.2026.102968

**Published:** 2026-06-18

**Authors:** Yu-ping Dong, Menglin Qiu, Haoyu Tang, Wen Shi, Yi Lu, Fang Ji, Hongwei Liao, Songmin Ying, Ping Zheng, Lin Wang

**Affiliations:** 1 State Key Laboratory of Genetic Evolution & Animal Models, Key Laboratory of Animal Models and Human Disease Mechanisms of Yunnan Province, Kunming Institute of Zoology, Chinese Academy of Sciences, Kunming, Yunnan 650201, China; 2 University of Chinese Academy of Sciences, Beijing 101408, China; 3 Department of Pharmacology & Department of Respiratory and Critical Care Medicine of the Second Affiliated Hospital, Zhejiang University School of Medicine, Key Laboratory of Respiratory Disease of Zhejiang Province, Hangzhou, Zhejiang 310009, China; 4 KIZ/CUHK Joint Laboratory of Bioresources and Molecular Research in Common Diseases, Kunming Institute of Zoology, Chinese Academy of Sciences, Kunming, Yunnan 650201, China

**Keywords:** human embryonic stem cell, early replication fragile sites, copy number variations, single-nucleotide variants, genomic stability

## Abstract

Long-term culture of human embryonic stem cells (hESCs) often induces chromosomal abnormalities, which limits their clinical use. However, the underlying mechanisms are unclear. Early replication fragile sites (ERFSs) are genomic loci susceptible to breakage in early S-phase and serve as hotspots for chromosomal rearrangements, with established links to carcinogenesis. To map ERFSs in hESCs, we established the early S-phase synchronization protocols and identified ERFSs. These ERFSs are enriched in GC content and short interspersed nuclear elements (SINEs) and are frequently located in promoters or enhancers of genes involved in pluripotency, proliferation, and genomic stability. ERFSs also overlap with regions associated with copy number variants (CNVs) and single nucleotide variants (SNVs) linked to cancers. Furthermore, we found that chromatin accessibility contributes to ERFS formation. Collectively, these findings provide a key resource for advancing ERFS research, offering insights into the phenotypic and genomic alterations observed in long-term hESC cultures.

## Highlights

• Genomic features of ERFSs in hESCs
• ERFSs in hESCs are predominantly located within regulatory genomic regions
• ERFSs are associated with genes regulating pluripotency/genomic stability
• ERFSs in hESCs are associated with cancer-related CNVs and SNVs

## Graphical abstract

**Figure:**
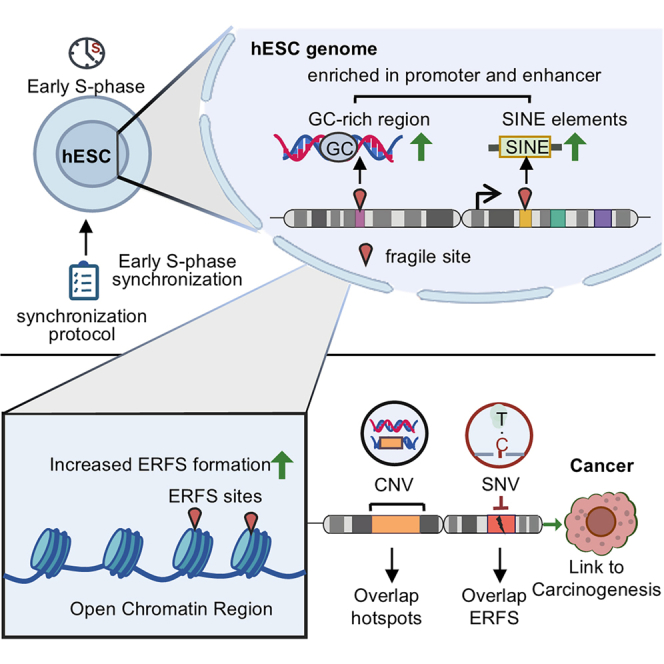

## Teaser

Long-term hESC culture causes chromosomal abnormalities, limiting clinical use. We mapped early replication fragile sites (ERFSs) in hESCs, which are enriched in GC content, SINE elements, and regulatory regions of pluripotency and genomic stability genes. ERFSs are associated with cancer-associated CNVs/SNVs and may be driven by chromatin accessibility, providing a key resource for understanding genomic instability.

## Introduction

Human embryonic stem cells (hESCs) are capable of self-renewal and differentiation into diverse cell types *in vitro*. Given these unique attributes, hESCs have served as physiologically relevant models for investigating developmental processes and disease mechanisms and hold great potential as cell sources for regenerative medicine and clinical therapies (Chong et al., 2014; Heidari Khoei et al., 2023; Reza et al., 2025; Yu et al., 2021).

However, large-scale translational applications of hESCs rely on long-term *in vitro* culture, a process that inevitably leads to the accumulation of genetic alterations, including copy number variations (CNVs), single-nucleotide variants (SNVs), and chromosomal numerical abnormalities (Draper et al., 2004; Hanson and Caisander, 2005; Lezmi et al., 2024; Narva et al., 2010). Such acquired mutations can compromise cellular proliferation and differentiation and may even predispose cells to tumorigenesis (Baker et al., 2007; Na et al., 2014). Despite the significant implications of these genetic changes, the mechanisms underlying their emergence in long-term cultured hESCs remain poorly understood.

Genomic instability in hESCs has often been linked to two major classes of fragile sites: common fragile sites (CFSs) and early replication fragile sites (ERFSs), both of which exhibit increased susceptibility to DNA breakage under replication stress. Le Tallec et al. reported that over 50% of recurrent deletions in cancer genomes arise from CFSs, which are preferentially located within large genes exceeding 300 kb in length (Le Tallec et al., 2013). Similarly, Barlow et al. identified ERFSs in B lymphocytes and demonstrated that more than half of recurrent amplifications and deletions in diffuse large B cell lymphoma originate from these sites, establishing ERFSs as another major source of cancer-associated genomic instability (Barlow et al., 2013). Mechanistically, CFSs typically reside in late-replicating genomic regions characterized by long AT-rich repeats, association with very large genes, and a tendency for incomplete replication—features that collectively contribute to their fragility under stress conditions (Debatisse and Rosselli, 2019; Ji et al., 2020). In contrast, ERFSs are organized in clusters within transcriptionally active regions, exhibit high GC content, and are often associated with gene-rich segments, distinguishing them from CFSs in both genomic distribution and sequence composition (Barlow et al., 2013).

A key unresolved question in hESC biology is which class of fragile sites—CFSs or ERFSs—serves as the primary driver of DNA damage and genomic alterations during long-term culture. Accumulating evidence suggests that hESCs display distinct mutational patterns compared to somatic cells, implying unique mechanisms of genomic instability. For instance, hESCs predominantly acquire uniparental disomy through chromosome loss and reduplication, whereas mitotic recombination—a common mutational source in somatic cells—is largely suppressed (Cervantes et al., 2002; Halliwell et al., 2020). Consistent with this, the rates of aberrant mitosis and chromosomal breaks per metaphase in human induced pluripotent stem cells are less than one-tenth of those in somatic cells (Kislova et al., 2023). Notably, many recurrently altered genes in long-term cultured hESCs—such as *MYC*, *FGFR3*, *ERBB2*, *ERBB3*, *NOTCH1*, and *TP53* (Lezmi et al., 2024)—are not large genes, which contrasts with the typical gene size associated with CFSs. Together, these observations suggest that CFSs are unlikely to be the main source of genetic variation in hESCs, raising the hypothesis that ERFSs may be the dominant contributors to genomic instability in long-term cultured hESCs. Testing this hypothesis requires mapping ERFSs in hESCs—a resource currently unavailable.

Previous studies achieved high-resolution mapping of ERFSs in B lymphocytes using high-throughput methods based on the colocalization of γH2AX, RPA, BRCA1, and SMC5 in early replication zones under hydroxyurea (HU)-induced replication stress (Barlow et al., 2013). However, this approach depends on robust cell synchronization protocols that are not directly applicable to hESCs, which are highly sensitive to conventional synchronization agents and prone to differentiation or cell death under suboptimal conditions. To date, the only small molecule shown to synchronize hESCs without altering their fundamental properties is nocodazole (Yiangou et al., 2019). Yet, because nocodazole arrests cells in M-phase, it cannot be directly used for ERFS mapping, which requires access to early S-phase cells.

To overcome this technical barrier, we developed a two-step synchronization strategy specifically designed for hESCs: cells were first synchronized in M-phase using nocodazole and then released to progress synchronously into early S-phase. Applying this optimized approach, we mapped hESC ERFSs by assessing the colocalization of γH2AX, RPA, BRCA1, and SMC5 in early replicating regions under HU-induced replication stress. Our analyses reveal that ERFSs are enriched within regulatory regions governing three core properties of long-term cultured hESCs: proliferation capacity, pluripotency maintenance, and genomic stability. These genomic hotspots may contribute to the accumulation of genetic variation during prolonged culture. These correlative findings provide a mechanistic framework for understanding endogenous genomic instability in hESCs and offer insights to optimize safer culture strategies for hESC-based applications.

## Results

### Genome-wide mapping of ERFSs in human embryonic stem cells

ERFSs are identified by treating early S-phase synchronized cells with HU. Specifically, ERFSs are defined as genomic regions where newly synthesized DNA—labeled with 5-ethynyl-2′-deoxyuridine (EdU)—is bound by DNA repair proteins such as BRCA1 and SMC5, as well as by replication protein A (RPA), a single-stranded DNA (ssDNA)-binding protein, during early S-phase (Barlow et al., 2013). To map ERFSs genome-wide in hESCs, we developed a two-step protocol to induce these sites and profile nascent DNA bound by DNA repair proteins and RPA during early S-phase.

**Figure 1:**
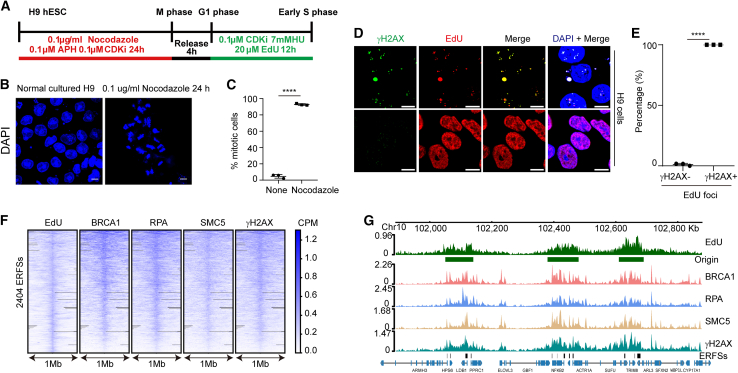
Genome-wide mapping of ERFSs in H9 hESCs (A) Schematic diagram of early S-phase synchronization for H9 hESCs. (B) M-phase synchronization validation by metaphase spread staining. Scale bars, 10 μm. (C) Quantification of the percentage of mitotic cells. (D) Images showing colocalization of EdU (red) with γH2AX protein (green) in nuclei after the cells were synchronized in early S-phase. Scale bars, 10 μm. (E) Quantification of the percentage of γH2AX foci that colocalized with EdU. (F) Heatmap of ERFS distribution on chromatin. ERFSs are identified by colocalization of EdU, BRCA1, RPA, SMC5, and γH2AX. (G) Gene tracks represent, from the top, EdU incorporation and bindings of BRCA1, RPA, SMC5, and γH2AX occupancy in chromosome 10 (q24.2 to q24.3 region). The *y* axis represents the counts per million (CPM). At least 50 cells were randomly analyzed in each replicate in (C and E). Experiments were repeated three times (*n* = 3), and similar results were obtained. Data were shown as mean ± SEM. Two-tailed Student’s *t* test, ∗∗∗∗*p* < 0.0001.

Under routine culture conditions, approximately 40%–65% of hESCs are in the S-phase (Becker et al., 2006). To avoid prolonged mitotic arrest and subsequent cell death, we employed a combination of nocodazole and low-dose treatments with aphidicolin (APH) and the CDK1 inhibitor (CDKi) RO-3306 to enhance cell synchronization efficiency during the first synchronization step (Figures S1A and S1B). Protocol optimization was performed using H9 hESCs (Figure 1A). Briefly, hESCs were synchronized in mitosis by treatment with 0.1 μg/mL nocodazole, 0.1 μM APH, and 0.1 μM CDKi for 24 h. Synchronization efficiency was confirmed by flow cytometry (Figures S2A and S2B) and microscopic examination (Figures 1B and 1C). Upon mitotic release, hESCs entered G1 phase within 4–5 h (Figure S2C). These cells were then treated with 7 mM HU for 12 h to achieve complete arrest at the G1/S transition (Figures 1D and 1E). During HU treatment and early S-phase synchronization, newly synthesized DNA was labeled with EdU (Figure 1A). Successful induction of ERFSs was supported by a high frequency of EdU-γH2AX colocalization, as shown by co-immunostaining (Figures 1D and 1E). Importantly, synchronization to early S-phase did not significantly affect global transcriptome (Figure S2D), chromatin accessibility (Figure S2E), and replication timing (Figures S2F and S2G).

We further tested whether this protocol could be applied to other hESC lines. We found that this protocol also effectively synchronized TJ-1# hESC to early S-phase with minor modification by changing the HU concentration from 7 to 1.8 mM in the second step (Figure S3A). Similar to H9 cells, TJ-1# cells achieve 70% arrest at the G1/S transition (Figures S3B–S3D) with a high frequency of EdU-γH2AX colocalization (Figures S3E–S3G). Additionally, the synchronization protocol for TJ-1# cells did not alter the global transcriptome (Figures S3H), chromatin accessibility (Figure S3I), and replication timing (Figures S3J–S3K).

For genome-wide mapping of ERFSs, G1/S-arrested cells were subjected to a Click-IT reaction to capture EdU-labeled genomic regions. In parallel, cleavage under targets and tagmentation (CUT&Tag) assays were performed to determine the binding sites of BRCA1, RPA, SMC5, and γH2AX. ERFSs were defined as genomic regions where EdU signals colocalized with these protein markers. Using this approach, we identified 2,404 high-confidence ERFSs in H9 cells (Figures 1F; Data S1), with representative loci depicted in Figure 1G. In TJ-1# cells, we identified 1,438 high-confidence ERFSs using the same method (Figure S4A; Data S1), with representative loci shown in Figure S4B. Importantly, 70% of ERFSs identified in TJ-1# cells overlapped with those in H9 cells (Figure S4C). Given that H9 cells are widely used in scientific research and have extensive multi-omics datasets available, we selected H9 cells as the model for subsequent downstream analyses.

### Distribution of ERFSs in hESCs

**Figure 2:**
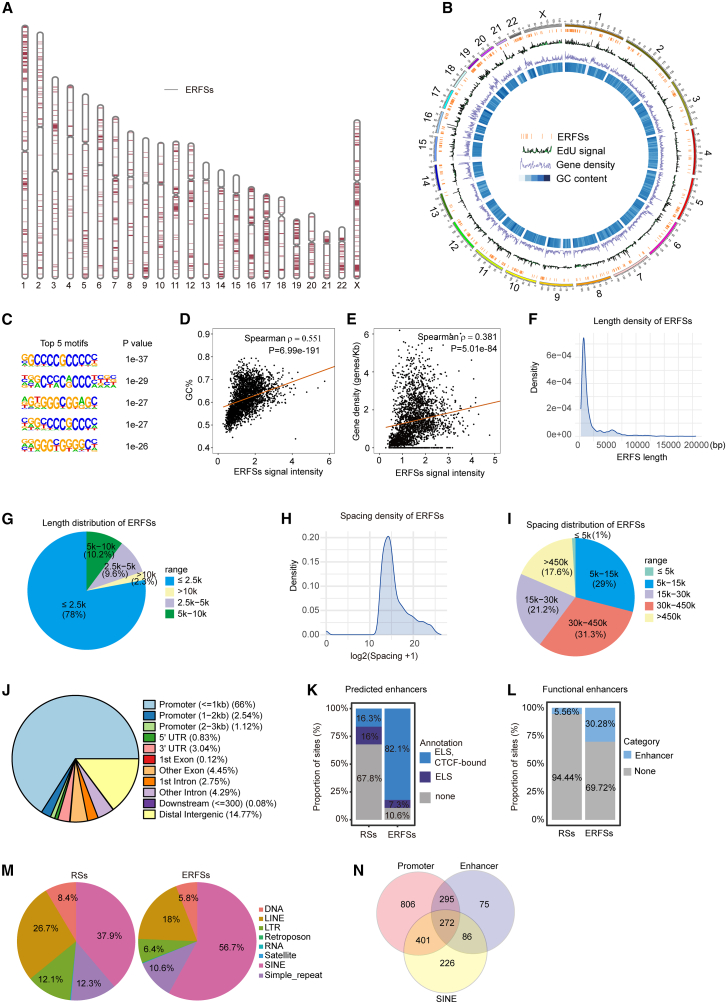
Genomic distribution of ERFSs in H9 hESCs (A) Chromosome view of the distribution of ERFSs. (B) Circos plot of the genomic locations of ERFSs, with EdU-seq signal, gene density, and GC content displayed by circles from outside to inside. (C) Top five motifs enriched in ERFSs. (D) Scatterplots illustrate the correlation between ERFSs and GC content. The signal intensity of ERFSs was represented by the mean intensity of four DNA damage response proteins (DDRPs), including RPA, SMC5, BRCA1, and γH2AX. (E) Scatterplots show the correlation between ERFSs and gene density. Consistent with panel (D), the signal intensity of ERFSs was represented by the mean intensity of the same four DDRPs (RPA, SMC5, BRCA1, and γH2AX). (F) Length density plot of ERFSs. (G) Length distribution of ERFSs. (H) Spacing density plot of ERFSs. (I) Spacing distribution of ERFSs. (J) Annotation of ERFSs using RefSeq genes. (K) ERFSs show a greater tendency to colocalize with enhancers than random sites (RSs). ELS, enhancer-like signatures. (L) ERFSs show a tendency to overlap with validated functional enhancers than RSs. (M) Compared with RSs, ERFSs are more prone to colocalize with SINEs. (N) Venn plot shows the relationship among ERFS overlapped promoters, validated enhancers, and SINE.

ERFSs were widely distributed across most human chromosomes (Figures 2A and 2B). ERFSs are enriched in GC content and (Figure 2C) showed a positive correlation with both GC content and gene density (Figures 2D and 2E), consistent with previous reports in somatic cells (Barlow et al., 2013). The sizes of ERFSs ranged from 0.2 to 20.6 kb, with the majority (78%) being shorter than 2.5 kb. The remaining ERFSs fell into the following size categories: 9.6% between 2.5 and 5 kb, 10.2% between 5 and 10 kb, and 2.3% longer than 10 kb (Figures 2F and 2G). The distances between adjacent ERFSs displayed a multimodal distribution: 29.0% were spaced 5–15 kb apart, 21.2% at 15–30 kb, 31.3% at 30–450 kb, and 17.6% beyond 450 kb (Figures 2H and 2I). This distribution revealed two distinct clusters of ERFS intervals: a dominant short-range group (<30 kb), accounting for 50.2% of all detected regions, and a long-range group (≥30 kb). This compact size is markedly narrower than the typical interval range reported in somatic cells (30–450 kb) and closely recapitulates the average replication fork spacing previously characterized in mouse embryonic stem cells (Ge et al., 2015). This consistency indicates that the compact genomic organization of hESC ERFS aligns with the dense pattern of replication initiation intrinsic to pluripotent stem cells.

To further characterize their genomic context, we annotated ERFSs using RefSeq. We found that 69% of ERFSs overlapped with gene promoters (Figure 2J), suggesting a potential role in transcriptional regulation. Comparative analysis with other genomic elements revealed that 89.0% of ERFSs co-localized with predicted enhancers (Figure 2K), 30% overlapped with validated enhancers (Figure 2L) and 56.7% overlapped with short interspersed nuclear elements (SINEs) (Figure 2M). Notably, 88% of validated enhancers (567/642) and 75% of SINE (673/899) overlap with promoters (Figure 2N; Data S2). Together, these results indicate that ERFSs are predominantly located within regulatory genomic regions, with strong enrichment at enhancer elements, supporting their potential involvement in transcriptional regulation.

### Association of ERFSs with genes implicated in pluripotency and genomic stability

Long-term culture of hESCs has been shown to promote proliferation while concurrently impairing differentiation capacity (Park et al., 2008). Supporting this, high-passage differentiated cells exhibit upregulation of undifferentiated markers, and teratomas derived from such cells demonstrate altered differentiation patterns compared to those from early-passage cultures (Xie et al., 2011). This progressive loss of pluripotency has been associated with mitochondrial dysfunction, characterized by elevated mitochondrial membrane potential, structural abnormalities, and increased production of reactive oxygen species (Xie et al., 2011).

**Figure 3:**
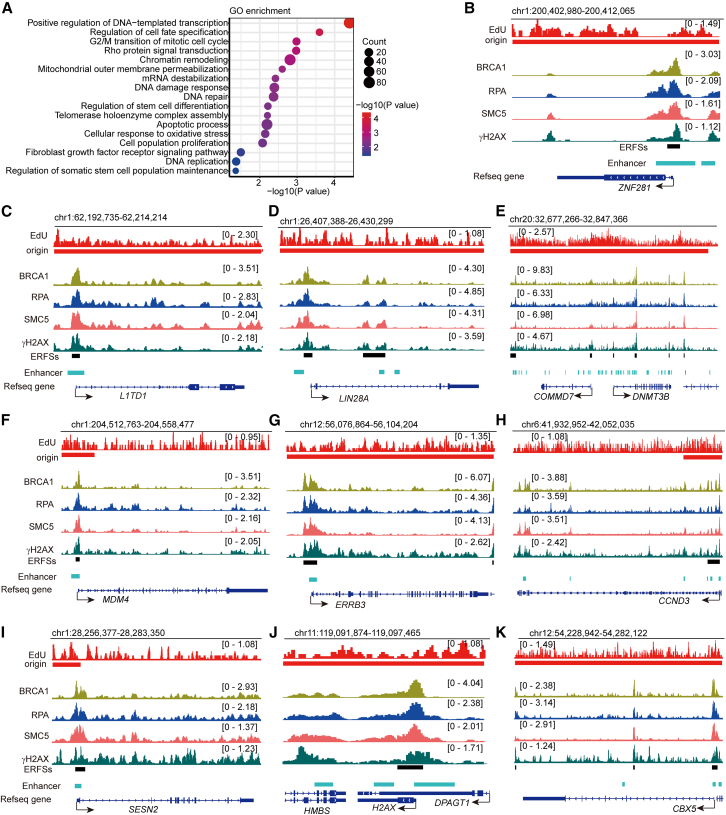
ERFSs may affect pluripotency, proliferation, and DNA damage response gene expression (A) Gene Ontology enrichment of genes associated with ERFSs. (B–K) Representative gene tracks were shown, including pluripotency genes (*ZNF281*, *L1TD1*, *LIN28A*, and *DNMT3B*), cell proliferation genes (*COMMD7*, *MDM4*, *ERBB3*, and *CCND3*), and DNA damage response genes (*SESN2*, *H2AX*, and *CBX5*).

To examine whether ERFSs are associated with these phenotypic changes, we identified genes linked to ERFSs and performed Gene Ontology (GO) analysis. The results revealed that ERFS-associated genes are involved in key biological processes related to hESC proliferation, pluripotency, and genomic stability. These include regulation of cell fate specification, G2/M transition of the mitotic cell cycle, Rho protein signal transduction, positive regulation of mitochondrial outer membrane permeabilization, DNA damage response, DNA repair, cell population proliferation, fibroblast growth factor receptor signaling, DNA replication, and regulation of somatic stem cell population maintenance (Figure 3A; Data S3).

We further analyzed the overlap between enhancers and ERFSs using validated hESC enhancer annotations (Barakat et al., 2018). In total, we identified 30 ERFS-overlapping enhancers linked to pluripotency-related genes and 73 such enhancers associated with genomic stability-related genes (Data S4). This genomic association was supported by genome browser visualization of representative loci, including pluripotency-related genes (e.g., *ZNF281*, *L1TD1*, *LIN28A*, and *DNMT3B*) (Figures 3B–3E), proliferation-related genes (e.g., *MDM4, ERBB3*, *COMMD7,* and *CCND3*) (Figures 3E–3H), and DNA damage response genes (e.g., *CBX5*, *H2AX*, and *SESN2*) (Figures 3I–3K). Collectively, these genomic analyses indicate that enhancers controlling pluripotency and genomic stability programs frequently coincide with ERFS, implying that these regulatory regions may be particularly susceptible to recurrent DNA breakage under replication stress during long-term hESC culture. Altered expression of these critical genes following enhancer damage represents a plausible mechanism that may contribute to the gradual functional decline observed in aged cultures; definitive functional validation of this cascade requires further experimental investigation.

### Association of ERFSs with copy number variations

**Figure 4:**
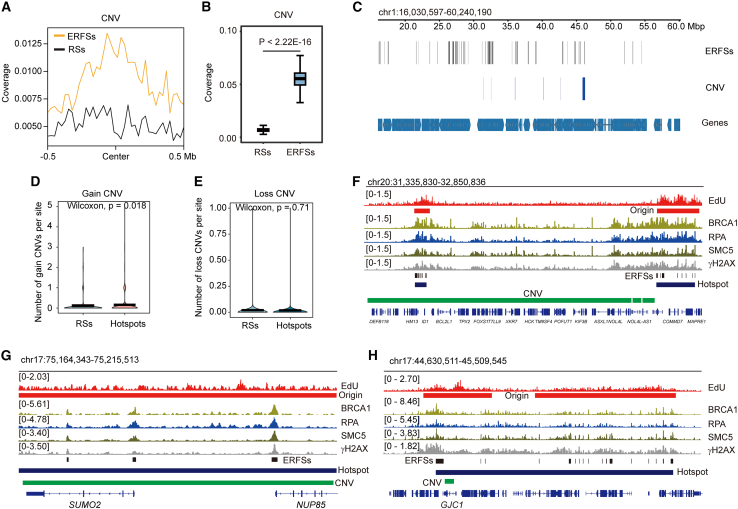
ERFSs are associated with gain of CNV (A) Aggregation plots displaying the distribution of CNVs in a 1-Mb window centered around the middle of ERFSs and random sites (RSs). The regions around ERFSs ±0.5 Mb were segmented into units of 25,000 bp, and the mean number of CNVs within each of these units were calculated. (B) Boxplot comparing the frequency of CNVs between ERFSs and RSs. (C) Representative CNV and ERFS loci are shown. (D) Violin plot showing the frequency of gain of CNVs between ERFS hotspots and RSs. (E) Violin plot showing the frequency of loss of CNVs between ERFS hotspots and RSs. (F–H) Genomic view of the representative hotspots’ loci. *p* values in (B, D, and E) were determined using the Wilcoxon rank-sum test.

To determine whether ERFSs are associated with genomic variation in hESCs, we analyzed whole-genome sequencing data from passage 59 H9 hESCs (Bernstein et al., 2010), identifying 476 copy number gains, 152 copy number losses, and 34,715 SNVs (Figure S5A; Data S5). We found that CNVs were preferentially located near the ERFSs compared with properly matched random regions derived exclusively from early-replicating genomic domains (Figures 4A and 4B). Furthermore, CNVs frequently co-occurred with clusters of ERFSs (Figure 4C). By integrating ERFSs clustered within 300 kb, we defined 291 ERFS hotspots (Data S6). These hotspots were strongly associated with copy number gains (Figure 4D) but not with copy number losses (Figure 4E). Notably, although most genomic regions showed no CNV overlap due to the rarity of such events, permutation and distance-based analyses confirmed that this association represents a robust biological signal rather than a statistical artifact of large sample size (Figures S5B and S5C). These CNV-associated ERFS hotspots encompassed 74 cancer-related genes (Data S7).

The link between ERFS hotspots and copy number gains was further corroborated by visual inspection of specific CNV loci, including 20q11.21 (Figure 4F), *SUMO2* (Figure 4G), and *GJC1* (Figure 4H). The 20q11.21 locus—recurrently reported in hESCs (Avery et al., 2013; Jeong et al., 2023; Lefort et al., 2008; Narva et al., 2010)—contains genes such as *ID1*, involved in tumor progression and ESC self-renewal (Li et al., 2017); *TPX2*, essential for mitotic survival (Kim et al., 2023); and *BCL2L1*, encoding the anti-apoptotic protein BCL-XL (Sillars-Hardebol et al., 2012). Amplification of this region, mediated by break-induced replication (Halliwell et al., 2020), enhances cell survival (Nguyen et al., 2014), impairs TGFβ-dependent neuroectodermal differentiation (Markouli et al., 2019), and has been linked to lung and gastric cancers (Jin et al., 2015; Tanikawa et al., 2018). The *SUMO2* locus includes the full-length *SUMO2* gene and part of *NUP85*. SUMO2, a ubiquitin-like modifier, plays a critical role in ESC fate determination (Borkent et al., 2016; Theurillat et al., 2020). The *GJC1* locus partially overlaps the *GJC1* gene, which encodes a gap junction protein that facilitates the generation of human induced pluripotent stem cells (Ke et al., 2017) and promotes proliferation in liver cancer cells (Chen et al., 2018).

We also identified ERFS hotspots at loci such as *AP4M1*, *GRID2*, and *CROCC* (Figures S5D–S5F), which are known to harbor frequent CNVs. Although these specific CNVs were not detected in our H9 passage 59 dataset, they have been previously reported in other hESC lines (Narva et al., 2010), underscoring the recurrent nature of genomic instability at these ERFSs.

### Association of ERFSs with cancer-related single-nucleotide variants

**Figure 5:**
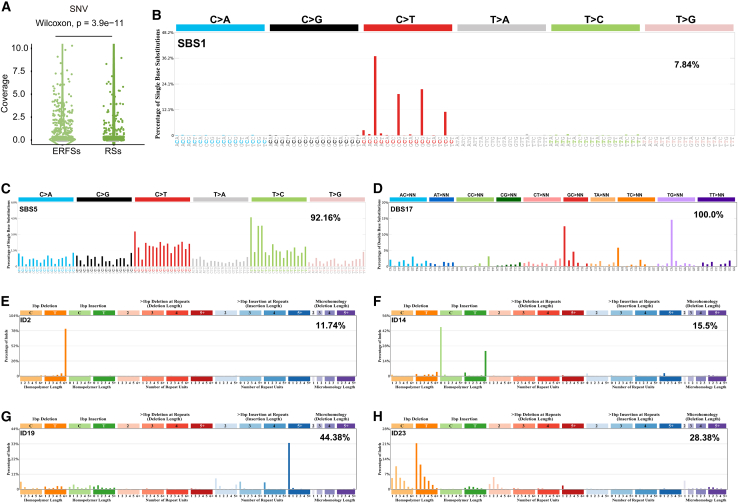
Classification of ERFSs associated SNVs (A) Scatterplot showing the frequency of SNVs between ERFS hotspots and RSs. *p* value was determined using the Wilcoxon rank-sum test. (B–H) SNV categories associated in ERFSs. SBS, single-base substitution; DBS, doublet base substitution; ID, small insertion or deletion.

SNVs arise from diverse endogenous and exogenous sources of DNA damage (Alexandrov et al., 2020). We, therefore, compared the SNV frequency in ERFSs versus random sites (RSs) and observed a significant association between ERFSs and SNV accumulation (Figure 5A).

Mutational signatures are closely linked to cancer development and subtypes (Alexandrov et al., 2013). To assess the potential oncogenic relevance of SNVs in hESCs, we analyzed base substitution patterns in H9 cells at passage 59 (Bernstein et al., 2010). Based on the COSMIC classification scheme, mutations were categorized into single-base substitutions (SBSs), doublet-base substitutions (DBSs), and small insertions or deletions (IDs). Our profiling revealed SBS1 and SBS5 as the predominant SBS types (Figures 5B and 5C), both of which are known to correlate with cell division rates (Alexandrov et al., 2015). The mutational burden of SBS5 was also positively associated with alterations in ERCC2 (Wijetunga et al., 2024). Among DBSs, DBS17 was the most frequent (Figure 5D)—a signature previously reported in breast cancer and a subset of lung cancers (Everall et al., 2026). The most common ID types included ID2, ID14, ID19, and ID23 (Figures 5E–5H). ID2 is widespread across multiple cancer types and tends to be highly elevated in cancer samples with defective DNA mismatch repair and microsatellite instability (Alexandrov et al., 2020), while ID19, primarily comprising 5-bp insertions, is observed in hematological malignancies and sarcomas (Everall et al., 2026). ID23 has been identified in renal cell carcinoma (Senkin et al., 2024). Together, these results suggest that the variant profiles in hESCs resemble cancer-associated mutational signatures, and that ERFSs may contribute to the acquisition of oncogenic mutations during stem cell propagation.

### Contribution of chromatin accessibility to ERFS formation

**Figure 6:**
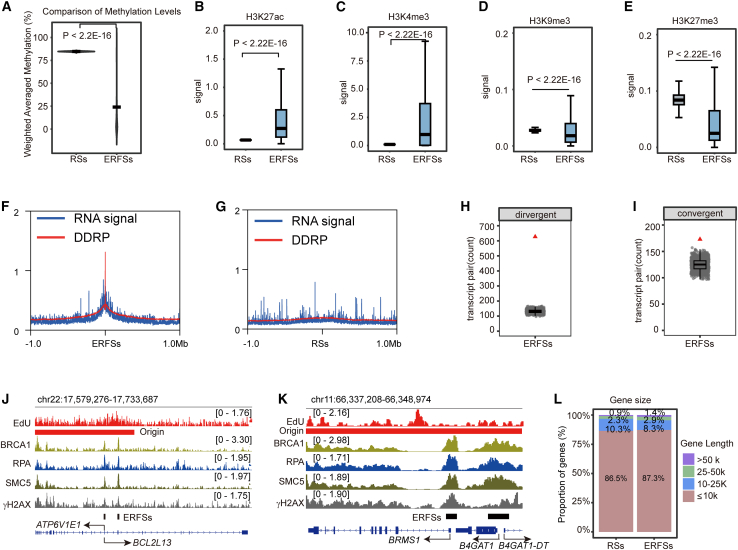
Chromatin accessibility contributes to ERFS formation (A–E) Quantitative comparison of the levels of DNA methylation, H3K27ac, H3K4me3, H3K9me3, and H3K27me3 between ERFSs and RSs, respectively. The solid black line represents the median, and the box denotes the interquartile range (IQR). *p* values were calculated using the Wilcoxon rank-sum test. (F and G) Aggregation plots of the distribution of the DDRP signal (red) and RNA-seq signal (blue) over ERFSs (F) or RSs (G) in a 2-Mb window centered on the midpoints of ERFSs or RSs. DDRP, DNA damage response protein. (H and I) ERFSs show a preferential localization near transcriptionally active divergent (H) and convergent (I) gene pairs in early S-phase. The count of such gene pairs overlapping ERFSs (indicated by red triangles) is assessed relative to a permutation-based background, which is represented by gray points. The boxplot summarizes the distribution of gene pair counts derived from the permutation model (*p* < 1 × 10^−3^). (J and K) Representative active divergent (J) and convergent (K) gene pairs are shown. (L) The fraction of RSs and ERFSs was analyzed in relation to gene size. Statistical significance was assessed by comparison with 1,000 iterations of randomly generated sites.

In somatic cells, ERFSs are enriched in highly transcribed regions and often reside between gene pairs arranged in convergent or divergent orientations (Barlow et al., 2013). To assess whether a similar pattern exists in hESCs, we first evaluated chromatin accessibility at ERFS regions using publicly available H9 cell datasets for DNA methylation and histone modifications, including H3K27ac, H3K4me3, H3K27me3, and H3K9me3 (Agostinho de Sousa et al., 2023). As expected, ERFSs exhibited significantly lower DNA methylation levels than RSs, along with markedly higher levels of the active marks H3K27ac and H3K4me3 (Figures 6A–6C), indicative of an open chromatin state permissive to transcription factor binding and active transcription. Consistently, the repressive marks H3K9me3 and H3K27me3 were substantially reduced at ERFSs relative to RSs (Figures 6D and 6E).

To further validate transcriptional activity associated with ERFSs, we performed RNA sequencing (RNA-seq) on H9 cells synchronized in early S-phase. ERFS regions showed a clear enrichment of RNA transcription signals compared to RSs (Figures 6F and 6G). Indeed, over 66% of RefSeq-annotated genes overlapping ERFSs were actively transcribed (fragments per kilobase of transcript per million mapped reads >1) (Data S8). Moreover, ERFSs were significantly enriched between gene pairs transcribed in convergent or divergent orientations (Figures 6H and 6I), as illustrated by the convergent pair *BCL2L13*/*ATP6V1E1* (Figure 6J) and the divergent pair *BRMS1*/*B4GAT1* (Figure 6K).

While multiple studies have linked large genes with CFSs (Gao et al., 2017; Helmrich et al., 2006; Maccaroni et al., 2020; Smith et al., 2006), we observed no significant difference in the frequency of large genes at ERFSs compared to RSs (Figure 6L). Together, these findings suggest that chromatin accessibility, rather than gene size, is more strongly associated with ERFS formation in hESCs, although this relationship remains a moderate correlative link and does not imply definitive causality.

## Discussion

Our study successfully established a landscape of ERFSs in hESC line, providing a valuable resource for the field. To enable analyses at biologically relevant scales, we defined ERFSs at two distinct resolutions: (1) typical ERFSs by merging peaks within 5 kb, following the canonical definition (Barlow et al., 2013), which was used for most genomic characterizations, and (2) ERFS hotspots by merging nearby ERFSs within 300 kb, a larger domain specifically used to analyze associations with large-scale copy number variations. Integrative analysis of ERFSs with other sequencing datasets revealed three key characteristics. (1) ERFSs in hESCs are predominantly located within regulatory genomic regions, with strong enrichment at enhancer elements. Specifically, they are closely associated with genes involved in pluripotency regulation and genomic stability maintenance, suggesting a functional role in modulating the expression of these genes. (2) ERFSs are significantly correlated with genomic variations such as CNVs and SNVs, implying that the fragility of these sites during early DNA replication may contribute to the formation of CNVs and cancer-associated SNPs. (3) ERFS formation is linked to chromatin accessibility.

The association between ERFSs and CNVs is of particular interest. CNVs represent a major form of genomic variation in long-term cultures. Previous studies have linked CNV hotspots to various fragile sites, such as AT-rich CFSs in human foreskin fibroblasts (Wilson et al., 2015), and tandem/G-rich or Alu repeats in the germline (Bose et al., 2014). Our data suggest that ERFSs may serve as primary drivers of copy number gains in hESCs. These ERFS-associated CNVs could further modulate the expression of genes regulating pluripotency, cell proliferation, and genomic stability, potentially influencing hESC survival and tumorigenic risk, thereby addressing a critical challenge in the clinical application of hESCs.

It should be noted that our analysis was limited to a single culture time point (passage 59 for H9). We cannot rule out the possibility that some mutation hotspots, particularly those resulting from gradual replication stress accumulation, may only become detectable after longer culture durations. Many reported hESC mutations, such as recurrent CNVs in pluripotency gene clusters, are known to emerge progressively over multiple passages (Kim et al., 2024), a dynamic that our single-time-point design could not capture. Consequently, our ERFS-based model does not fully explain all previously reported hESC mutations. Future studies should incorporate whole-genome sequencing data from multiple time points to enable comprehensive analysis and validation.

In summary, our refined ERFS map offers a high-resolution resource for deciphering the molecular mechanisms underlying genomic instability in hESCs. Beyond advancing the understanding of ERFS biology, this resource may guide functional studies targeting ERFS-associated genes and inform the optimization of hESC culture conditions—for instance, by mitigating replication stress at ERFSs. Ultimately, these efforts will help support the safe and effective use of hESCs in regenerative medicine and disease modeling.

## Resource availability

### Lead contact

Further information and requests should be directed to Dr. Lin Wang (wanglin2015@mail.kiz.ac.cn).

### Materials availability

This study did not generate any unique cell lines or use any unique materials or reagents.

### Data and code availability

All of the sequencing data have been deposited in the National Genomics Data Center database with the following accession number: HRA013289 (https://ngdc.cncb.ac.cn/gsa-human/browse/HRA013289). This study does not report original code.

## Acknowledgments

This work was supported by National Key Research & Developmental Program of China (2021YFA1102000), Yunnan Province funding (202305AH340006), and Yunnan Revitalization Talent Support Program Young Talent Project to L.W.; 10.13039/501100002367CAS “Light of West China” Program to L.W.; Yunnan Fundamental Research Projects (grant no. 202301AS070062); and Yunnan Fundamental Research Projects (202401AW070009).

## Author contributions

Y.-p.D. and L.W. performed most of the experiments; W.S. performed FACS experiments; Y.L. performed the EdU-seq; M.Q. and H.T. performed bioinformatics analysis; F.J., H.L., S.Y., and P.Z. discussed the project; L.W. designed the experiments, interpreted the results, and wrote the original manuscript; and P.Z. revised the manuscript.

## Declaration of interests

The authors declare no competing interests.

## Declaration of generative AI and AI-assisted technologies in the writing process

Generative AI (Doubao) was used to assist with language polishing and readability improvement. All authors have reviewed and revised the full content of the manuscript and take full responsibility for its accuracy and integrity.

## STAR★Methods

### Key resources table

**Table:** 

REAGENT or RESOURCE	SOURCE	IDENTIFIER
**Antibodies**		

γ-H2AX for immunofluorescence	Cell signaling	Cat# 80312; RRID: AB_2799949
γ-H2AX for CUT&Tag	Merk-millipore	Cat# 05-636-I; RRID: AB_2755003
RPA	Abcam	Cat# ab240637; RRID: AB_3750734
SMC5	Proteintech	Cat# 14178-1-AP; RRID: AB_2192775
BRCA1	Beyotime	Cat# AF6339; RRID: AB_3750735
Alexa Fluor 488-conjugated goat anti-mouse IgG (H + L) secondary antibody	Thermo Fisher Scientific	Cat# A-11029; RRID: AB_2534088

**Chemicals, peptides, and recombinant proteins**		

hESC-Qualified Matrix, LDEV-free	Corning	Cat# 354277
KnockOut Serum Replacement	Thermo Fisher	Cat# 10828028
Dimethyl sulfoxide	Beyotime	Cat# ST038
Nocodazole	MedChemExpress	Cat# HY-13520
Aphidicolin	Abcam	Cat# ab142400-1mg
RO-3306	MedChemExpress	Cat# HY-12529
Hydroxyurea	Sigma	Cat# H8627-5G
Accutase	Sigma	Cat# A6964
RNase A	Beyotime	Cat# C1008M
5-Ethynyl-2′-deoxyuridine	Beyotime	Cat# C0075S
DAPI	Thermo Fisher	Cat# D1306
CuSO4	Sigma	Cat# 451657
Sodium ascorbate	Sigma	Cat# A4034
biotin-azide	Thermo Fisher	Cat# B10184
Y-27632	MedChemExpress	Cat# HY-10071
Dynabeads MyOne Streptavidin C1	Thermo Fisher	Cat# 65001
AMPure XP	Beckman Coulter	Cat# A63880
TRNzol	Tiangen	Cat# DP424
hESC culture medium	Cauliscell	Cat# 400105

**Critical commercial assays**		

Hyperactive Universal CUT&Tag Assay Kit for Illumina Pro	Vazyme	Cat# TD903
TruePrep Index Kit V2 for Illumina	Vazyme	Cat# TD202
KAPA HyperPrep Kit	KAPA	Cat# KK8502
Hieff NGS® Ultima Dual-mode RNA Library Prep Kit	Yeasen	Cat# 12308ES24
Hyperactive ATAC-Seq Library Prep Kit for Illumina	Vazyme	Cat# TD711

**Deposited data**		

Raw sequencing data	This paper	GSA: HRA013289
Human reference genome, GRCh38/hg38	Genome Reference Consortium	https://hgdownload.soe.ucsc.edu/goldenPath/hg38/
Whole Genome Sequencing data	Bernstein et al. (2010)	GEO: GSM1227088
Published functional enhancer datasets used in this study	Barakat et al. (2018)	GEO: GSE99631
Predicted enhancers	ENCODE	https://www.encodeproject.org
Repeat sequences data	UCSC Genome Browser	http://genome.ucsc.edu/
DNA methylation data	Agostinho de Sousa et al. (2023)	GEO: GSM6749234
Histone modification ChIP-seq data	Agostinho de Sousa et al. (2023)	GEO: GSE218510

**Experimental models: Cell lines**		

H9 embryonic stem cells	N/A	Provided by Professor Lei Li from Institute of Zoology, Chinese Academy of Sciences
TJ-1# embryonic stem cells	Bi et al. (2020)	Provided by Professor Yixuan Wang from Tongji university

**Software and algorithms**		

Trim Galore	Babraham Bioinformatics	https://github.com/FelixKrueger/TrimGalore; RRID:SCR_011847
Bowtie2	Langmead and Salzberg (2012)	http://bowtie-bio.sourceforge.net/bowtie2/index.shtml; RRID:SCR_016368
SAMtools	Danecek et al. (2021)	https://www.htslib.org/; RRID:SCR_002105
MACS2	Zhang et al. (2008)	https://pypi.org/project/MACS2/; RRID:SCR_013291
bedtools	Quinlan and Hall (2010)	https://github.com/arq5x/bedtools2; RRID:SCR_006646
pybedtools	Dale et al. (2011)	https://daler.github.io/pybedtools/#; RRID:SCR_021018
ChIPseeker	Yu et al. (2015)	https://bioconductor.org/packages/ChIPseeker/; RRID:SCR_021322
DAVID tool	Sherman et al. (2022)	https://david.ncifcrf.gov/; RRID:SCR_001881
HISAT2	Kim et al. (2019)	http://ccb.jhu.edu/software/hisat2/index.shtml; RRID:SCR_015530
deepTools	Ramirez et al. (2014)	https://deeptools.readthedocs.io/en/develop; RRID:SCR_016366
Burrows Wheeler Aligner	Li and Durbin (2009)	http://bio-bwa.sourceforge.net/; RRID:SCR_010910
GATK HaplotypeCaller	McKenna et al. (2010)	https://gatk.broadinstitute.org/hc/en-us; RRID:SCR_001876
GATK VariantFiltration	McKenna et al. (2010)	https://gatk.broadinstitute.org/hc/en-us; RRID: SCR_028441
Control-FREEC	Boeva et al. (2012)	http://bioinfo-out.curie.fr/projects/freec/tutorial.html; RRID:SCR_010822
SigProfilerMatrixGenerator	Bergstrom et al. (2019)	https://github.com/AlexandrovLab/SigProfilerMatrixGenerator/; RRID:SCR_023122
SigProfilerExtractor	Islam et al. (2022)	https://github.com/AlexandrovLab/SigProfilerExtractor/; RRID:SCR_023121
CoolBox	Xu et al. (2021)	https://github.com/GangCaoLab/CoolBox; RRID:SCR_023121
Integrative Genomics Viewer	Robinson et al. (2011)	http://www.broadinstitute.org/igv/; RRID:SCR_011793
Circos	Krzywinski et al. (2009)	http://circos.ca/; RRID:SCR_011798
ggplot2	Wickham (2016)	https://cran.r-project.org/web/packages/ggplot2/index.html; RRID:SCR_014601
CCIVR	Ohhata et al. (2022)	https://github.com/CCIVR/ccivr; RRID: RRID:SCR_028426
bedGraphToBigWig	UCSC Genome Browser	https://genome.ucsc.edu/goldenpath/help/bigWig.html; RRID:SCR_028439
GraphPad Prism GraphPad Software V.8	N/A	http://www.graphpad.com/; RRID:SCR_002798

### Experimental model and study participant details

This study used established human embryonic stem cell lines and did not recruit living human participants. Two hESC lines, one male (TJ-1#) and one female (H9), were included. This study was not statistically powered nor experimentally designed to rigorously evaluate sex-associated differences in experimental outcomes. Consequently, sex-dependent effects could not be systematically elucidated, which constitutes a limitation to the generalizability of our findings beyond the two specific cell lines examined herein.

### Method details

#### hESC culture and cell cycle synchronization

Human primed ESCs with H9 background was kindly provided by Professor Lei Li from Institute of Zoology, Chinese Academy of Sciences. TJ-1# human primed ESCs was derived by Tongji Hospital (Bi et al., 2020) and kindly provided by Professor Yixuan Wang from Tongji university. hESCs were maintained in TeSR-E8 medium on Matrigel (Corning, 354277)-coated dishes (Corning) at 37°C, 5% CO2. The medium was refreshed daily, and the cells were passaged every 5 days with the split ratio of 1:6 using 0.5 mM EDTA (pH 8.0). H9 cells were utilized between passages 60 and 75, and TJ-1# cells were used within the passage range of 25 to 35. For cryopreservation, hESCs were frozen in a solution consisting of 90% KnockOut Serum Replacement (Thermo Fisher) supplemented with 10% DMSO and stored in liquid nitrogen until thawing. Mycoplasma testing was performed weekly to ensure the cells remained contamination-free.

To synchronize hESCs in the early S-phase of the cell cycle, the following steps were performed: Cells were first synchronized in the M phase by treatment with 100 ng/mL nocodazole (MedChemExpress, HY-13520), 0.1 μM aphidicolin (Abcam, ab142400-1mg), and 0.1 μM RO-3306 (MedChemExpress, HY-12529) for 24 hours. Subsequently, cells were washed three times with PBS and released into the G_0_/G_1_ phase by incubation in pre-warmed medium for 4 hours. Finally, cells were synchronized in the early S-phase by treatment with 0.1 μM RO-3306 combined with 7 mM hydroxyurea (HU) for H9 cells or 1.8 mM HU for TJ-1# cells, followed by 12 hours of additional treatment.

#### Cell cycle profile analysis

Cells at different time points during synchronization were dissociated into single cells using Accutase (Sigma, A6964) and fixed in ice-cold 70% ethanol at 4°C overnight. After washing 3 times with PBS, cells were treated with 100 μg/mL RNase A and stained with 10 μg/ml propidium iodide (Beyotime, C1008M) for 30 minutes, separately. Cell cycle analysis was performed using a BD LSRFortessa™ flow cytometer, and data were analyzed with FlowJo software.

#### Immunofluorescence

Cells were seeded on Matrigel coated glass coverslips and incubated with 20 μM 5-Ethynyl-2′-deoxyuridine (EdU, Beyotime, C0075S) during early S-phase synchronization. After fixation with 4% paraformaldehyde for 15 min at room temperature, cells were washed three times with PBS. Cells were then permeabilized with 0.1% Triton X-100 at 4°C for 15 min, followed by incubation with the Click-iT reaction cocktail for 30 min. For γH2AX staining, cells were blocked in 5% BSA in PBS for 1 hours at room temperature, incubated with γH2AX mouse primary antibody (Cell Signaling Technology, #80312, 1:1000) overnight at 4°C, and subsequently incubated with Alexa Fluor 488-conjugated goat anti-mouse IgG (H+L) secondary antibody (Thermo Fisher Scientific, A-11029, 1:500) for 1 hour. Finally, cells were counterstained with DAPI (Thermo Fisher Scientific) and examined using Olympus FV1000 confocal microscope.

#### CUT&Tag and data processing

Cleavage under targets and tagmentation (CUT&Tag) experiments were constructed using the Hyperactive Universal CUT&Tag Assay Kit for Illumina Pro (Vazyme, TD903) following the manufacturer’s protocol. Briefly, 1 × 10^5^ cells were harvested and resuspended in a mixture containing concanavalin A-coated beads and primary antibody, followed by incubation at 4°C overnight. After removing the mixture and washing, cells were incubated with secondary antibody for 1 hour at room temperature with gentle rotation. Cells were then washed using dig-wash buffer and incubated with the pA-Tn5 adapter complex for 1 hour at room temperature with gentle rotation. Following tagmentation, genomic DNA was extracted and used for library construction with the TruePrep Index Kit V2 for Illumina (Vazyme, Cat. No. TD202). CUT&Tag libraries were sequenced on NavaSeq6000 platform. Experiments were repeated at least three times.

Raw CUT&Tag reads were first trimmed to remove adapter sequences using Trim Galore (version 0.6.10) with the following parameters: -q 25 --phred33 --length 25 -e 0.1 --stringency 4, followed by quality assessment. The cleaned reads were then aligned to the human reference genome (GRCh38) using Bowtie2 (version 2.5.1) (Langmead and Salzberg, 2012) with default parameters. Aligned reads in sorted BAM format were filtered for high mapping quality and deduplicated using SAMtools (version 1.19.2) (Danecek et al., 2021). Then, BAM files from each group were combined, and peak calling was performed using MACS2 (version 2.2.9.1) (Zhang et al., 2008) to identify significantly enriched genomic regions. Peaks located within 5 kb of each other were subsequently merged to define broader enriched domains.

#### Early replication initiation zones definition

Cells synchronized in early S-phase were fixed in 90% ice-cold methanol on ice for 20 min and permeabilized with 0.5% Triton X-100 in PBS for 20 min. Cells were resuspended in a fresh prepared biotin-azide click cocktail [100 mM Tris, pH 8.0, 100 mM CuSO4 (Sigma), 100 mM sodium ascorbate (Sigma, A4034), 10 mM biotin-azide (Thermo Fisher, B10184)], incubated at 37°C, 30 min. Cells were subsequently lysed in lysis buffer (10 mM Tris-HCl, pH 8.0, 0.5% SDS, 0.2 mg/mL Proteinase K) at 50°C for at least 3 h. Genomic DNA was extracted using phenol/chloroform, resuspended, and sonicated to an average fragment size of 300–400 bp using an ultrasonic cell disruptor (Xiaomei). Biotinylated DNA was enriched with Dynabeads MyOne Streptavidin C1 (Thermo Fisher, 65001). Libraries were constructed using KAPA HyperPrep Kit (KK8502) according to the manufacturer’s instructions, followed by purification with AMPure XP (Beckman Coulter, A63880). Libraries were quantified and sequenced by Annoroad Gene Technology Co., Ltd. (Beijing, China) on the NovaSeq X Plus platform.

To identify early replication initiation zones (ERIZs), the genome was divided into non-overlapping 10 kb bins. The number of EdU-seq reads in each bin was counted and normalized to counts per million (CPM). Background signals were subtracted using control input. Adjacent bins with enriched signals located within 50 kb of each other were merged using bedtools merge function (v2.30.0) (Quinlan and Hall, 2010) to define the final early replication initiation zones (ERIZs).

#### Identification of ERFSs and random sites

Genomic regions co-occupied by SMC5, RPA, and BRCA1 were identified using pybedtools (version 0.10.0) by intersecting their individual peak sets. Following the strategy described in the original ERFS study (Barlow et al., 2013), adjacent co-occupied peaks within 5 kb were merged. These regions were further supported by γH2AX signal, indicating activation of the DNA damage response at these loci. Only regions located within ERIZs were retained and defined as early replicating fragile sites (ERFSs). For comparison, random sites (RSs) were generated from early-replicating regions after excluding blacklist regions, with size and number matched to those of ERFSs on each chromosome.

#### Identification of ERFS hotspots and random sites

Given that CNVs encompass large genomic regions, to better characterize the relationship between ERFSs and CNVs, ERFSs clustered within a 300 kb window, as described by Barlow et al. (2013), were merged and defined as ERFS hotspots. For comparison, random sites were generated from early-replicating regions after excluding blacklist regions, with size and number matched to those of ERFS hotspots on each chromosome.

#### ERFS motif enrichment and correlation with GC content and gene density

Motif enrichment analysis of H9 ERFSs was performed using the findMotifsGenome.pl module from HOMER v5.1 (Heinz et al., 2010). Correlations of ERFSs with GC content and gene density were analyzed using Spearman’s correlation test in R. The signal intensity of ERFSs was defined as the mean intensity of four DNA damage response proteins (DDRPs), including RPA, SMC5, BRCA1, and γH2AX.

#### ERFSs annotations and gene ontology analysis

ERFSs of H9 were annotated to their closest genes using the R package ChIPseeker (version 1.34.1) (Yu et al., 2015), with gene annotations from the RefSeq database. Gene ontology (GO) enrichment analysis was conducted using the online DAVID tool (Database for Annotation, Visualization and Integrated Discovery) with default settings (Sherman et al., 2022).

#### RNA extraction and RNA-seq data analysis

Total RNA was extracted using TRNzol (Tiangen, Cat. No. DP424) following the standard protocol. Strand-specific libraries were constructed with the Hieff NGS® Ultima Dual-mode RNA Library Prep Kit and sequenced on the NovaSeq 6000 platform. Raw RNA-seq reads were first trimmed using Trim Galore with default parameters, then aligned to the human genome (hg38) using HISAT2 (Kim et al., 2019). Fragments per kilobase of transcript per million mapped reads (FPKM) were calculated using Cufflinks. For ERFSs and RSs, the reads were counted by multibamSummary. The fragments per kilobase per million mapped reads (FPKM) were calculated to represent the transcription signal for ERFSs and RSs.

#### ATAC-seq and data analysis

ATAC-seq libraries were prepared using the Hyperactive ATAC-Seq Library Prep Kit for Illumina (Vazyme, TD711) according to the manufacturer’s instructions. In brief, 1 × 10^5^ cells were harvested, washed twice with 50 μL ice-cold TW buffer, and lysed in 50 μL ice-cold lysis buffer for 5 min. Nuclei were then pelleted by centrifugation at 500 × g for 10 min at 4°C. Following removal of the supernatant, the nuclear pellet was subjected to Tn5 transposase fragmentation at 37°C for 30 min. After terminating the reaction with stop buffer, DNA was purified using ATAC DNA extraction beads, followed by PCR amplification and size selection using ATAC DNA clean beads.

ATAC-seq reads were first trimmed to remove adapter sequences using Trim Galore (version 0.6.10), followed by quality assessment. Cleaned reads were aligned to the GRCh38 reference genome using Bowtie2 (version 2.5.1). The resulting BAM files were sorted and filtered to retain high-quality alignments, while mitochondrial reads were removed and PCR duplicates were excluded using SAMtools (version 1.19.2). Genome-wide signal tracks were generated in BigWig format with CPM normalization using bamCoverage (deepTools version 3.5.1). Correlations between ATAC-seq samples were assessed using multiBamSummary and plotCorrelation (deepTools version 3.5.1).

#### Synchronization effects on transcriptome and chromatin accessibility

To evaluate the potential impact of cell synchronization, we assessed global transcriptomic and chromatin accessibility profiles. Sample correlations were calculated using multiBamSummary and plotCorrelation.

#### Validation of replication timing in synchronized cells

To verify the replication timing of ERFSs identified in synchronized cells, we employed two complementary approaches using asynchronous hESCs. First, we analyzed EdU-seq from cells sorted into early-S and late-S phase fractions by fluorescence-activated cell sorting (FACS). EdU signal intensity over the defined ERFS intervals was quantified using multiBigwigSummary (version 3.5.5) in BED-file mode, and the enrichment of signals in early-S versus late-S fractions was visualized using scatter plots. Second, we compared the drug-induced ERFSs with physiological early-replicating regions defined by the S50 replication timing estimator (Dellino et al., 2013). Genomic overlaps were computed using bedtools intersect and visualized as a Venn diagram.

#### Correlation of ERFS with convergent/divergent transcripts

Annotated genes from Gencode v47 located on opposite DNA strands were classified as convergent transcripts if their transcription end sites (TES) were within 5 kb of the opposite flanks of an ERFS center, or if their intragenic regions overlapped. Divergent transcript pairs were defined as transcripts on opposite strands whose transcription start sites (TSS) were within 5 kb of the opposite flanks of an ERFS center. The counts of divergent and/or convergent transcript pairs overlapping ERFS were quantified using ccivr (version 2.0) and compared to corresponding RSs.

#### Identification of SNV and CNV

Whole genome sequencing data of H9 cells, cultured in KO DMEM supplemented with 20% KSR and bFGF (10ng/mL), were retrieved from the GEO database (accession number: GSM1227088). Reads were trimmed using Trim Galore with default settings and mapped to the human genomes (hg38) by the Burrows Wheeler Aligner (BWA) (Li and Durbin, 2009). Duplicate marking and local realignment around indels were performed using SAMtools. SNVs and indels were called using GATK HaplotypeCaller. To obtain high-quality SNVs and indels, we first applied filtration using GATK VariantFiltration with the following criteria: “QD < 2.0 || MQ < 40.0 || FS > 60.0 || SOR >3.0 || (vc.hasAttribute(‘MQRankSum’) && MQRankSum < -12.5) || (vc.hasAttribute(‘ReadPosRankSum’) && ReadPosRankSum < -8.0) || GQ < 60”. Variants passing this initial filtration were further retained based on the following criteria: (i) each variant site must be covered by at least 20 reads; (ii) each variant must be supported by at least 5 reads, with the ratio of forward reads to total reads ranging from 0.3 to 0.7. Estimated copy numbers were inferred using Control-FREEC with the default configuration file. Copy number variations (CNVs) were filtered out if their estimated copy numbers fell outside the range of 1 to 3. For CNV coverage profiling, CNV coordinates were converted to BigWig format using bedGraphToBigWig. deepTools was then used to quantify and visualize CNV coverage centered on ERFSs and random sites (RSs) within a ±0.5 Mb window. SNV frequency for each ERFS and its corresponding control site was represented by the number of SNVs per megabase (SNVs/Mb).

#### Analysis of ERFS hotspot and CNV overlap

The observed percentage of ERFS hotspots overlapping CNV gains or losses was determined using bedtools intersect. A null distribution was constructed from 1,000 randomized genomic sets, strictly matched for hotspot number and size. Empirical *p* values represent the proportion of permutations with overlaps exceeding the observed values.

#### Evaluation of proximity to CNV gains

To evaluate spatial proximity, the linear genomic distances from each CNV gain to the nearest ERFS hotspot or random site were calculated using bedtools closest (with the -d flag), and the resulting distributions were compared using a Wilcoxon rank-sum test.

#### SNV classification

SNV signatures were discovered using the SigProfiler tool suite with default standard parameters. SigProfilerMatrixGenerator (Bergstrom et al., 2019) was employed to construct mutational matrices, which incorporated somatic mutations along with their adjacent sequence context. Subsequently, the SBS96, DBS78, and ID83 matrices were used as inputs for SigProfilerExtractor (Islam et al., 2022) to perform *de novo* extraction of mutational signatures. These extracted signatures were further deconvoluted against the published COSMIC v.3.2 signatures.

#### Correlation analysis of ERFSs and enhancers

Enhancer-like signatures (ELS) and CTCF-bound ELS annotations were obtained from ENCODE (https://www.encodeproject.org). Validated functional enhancers in hESCs were from Barakat et al. (2018); Barakat et al. (2018). Overlaps between ERFSs and these enhancer elements, as well as between ERFSs and their corresponding control sites, were computed via bedtools closest function. Proportions of regions overlapping with ELS, CTCF-bound ELS, or lacking enhancer features were visualized as stacked bar charts.

#### Correlation analysis of ERFSs and repeat sequences

The annotation of repeat sequences, including Alu elements, LINEs and SINEs, was obtained from the UCSC genome database (http://genome.ucsc.edu/), their abundance in each ERFS or RS was then calculated using R.

#### Correlation analysis of ERFSs and DNA methylation

DNA methylation data were retrieved from the GEO database (accession number: GSM6749234). Methylation coverage files were processed to compute methylation levels at each genomic site. The methylation levels of ERFSs and their corresponding randomly generated control sites were determined using the bedtools intersect function.

#### Correlation analysis of ERFSs and histone modification

Histone modification ChIP-seq data were obtained from the GEO database (accession number GSE218510). After quality assessment, raw reads were trimmed for adapters and low-quality bases using Trim Galore, then aligned to the human GRCh38 reference genome using Bowtie2 (v2.5.1). Resulting BAM files were sorted, filtered for high-quality mapped reads, and deduplicated via SAMtools (v1.19.2). deepTools2 (version 3.5.5) was used to generate BigWig-formatted normalized coverage tracks (normalized by CPM). Histone modification levels were quantified at ERFSs and their corresponding control sites.

#### Data visualization

All the representative genomic profiles were drawn using CoolBox (version 0.3.9) (Xu et al., 2021) or Integrative Genomics Viewer (IGV). The Circos plots were made using Circos (version 0.69.8) (Krzywinski et al., 2009) to present the whole genome, and the aggregation plots were drawn using deepTools2 (version 3.5.5). Data visualization was performed using ggplot2 (version 3.5.1) in R, with DNA methylation patterns displayed as violin plots, histone modification levels as boxplots, and gene size distributions as bar plots.

### Quantification and statistical analysis

Relevant statistical information, such as sample sizes, definitions of central tendency and dispersion measures, and exact P-values, is specified in figure legends, the main text, and STAR Methods subsections. A consolidated overview of the analytical strategies is presented below.

**Table:** 

Experiment	Software	Statistical test or model	Sample size	Measures of center ± dispersion	Location
Figures 1C and 1E	Prism 8	Two-tailed Student’s *t* test	At least 50 cells in each replicate. Experiments were repeated three times.	Mean ± s.e.m.	Figures 1C and 1E legend
Figure 2C	Homer	Motif enrichment analysis	All identified ERFSs in H9	–	Method: “ERFSs annotations and gene ontology analysis”
Figures 2D and 2E	R	Spearman correlation	All identified ERFSs in H9	–	Method: “ERFS motif enrichment and correlation with GC content and gene density”
Figure 3A	DAVID	Modified Fisher’s exact test	ERFS-associated genes	–	Method: “ERFSs annotations and gene ontology analysis”
Figure 4B	R	Two-sided Wilcoxon rank-sum test	All ERFSs and RSs identified in H9	Median with IQR	Method: “identification of SNV and CNV”
Figures 4D and 4E, and 5A	R	Two-sided Wilcoxon rank-sum test	All ERFSs and RSs identified in H9	Mean and data distribution	Method: “identification of SNV and CNV”
Figure 6A	R	Two-sided Wilcoxon rank-sum test	All ERFSs and RSs identified in H9	Mean and data distribution	Method: “correlation analysis of ERFSs and DNA methylation”
Figures 6B–4E	R	Two-sided Wilcoxon rank-sum test	All ERFSs and RSs identified in H9	Mean and data distribution	Method: “correlation analysis of ERFSs and histone modification”
Figures 6H and 6I	R	Permutation model	All ERFSs and RSs identified in H9	Mean	Method: “correlation of ERFS with convergent/divergent transcripts”
Figures S2D, S2E, S3H, S3I	DeepTools	Pearson correlation	2/3	–	Method: “synchronization effects on transcriptome and chromatin accessibility”; Figures S2 and S3 legend
Figure S3F	Prism 8	Mann–Whitney U test	20	Mean ± SD	Figure S3F legend
Figure S5B	R	Permutation model	All ERFSs and RSs identified in H9	Mean	Method: “analysis of ERFS hotspot and CNV overlap”
Figure S5C	R	Two-sided Wilcoxon rank-sum test	All ERFSs and RSs identified in H9	Mean and data distribution	Method: “evaluation of proximity to CNV gains”

## Supplemental information

Document S1. Figures S1–S5

Data S1. ERFS sites

Data S2. ERFS-overlapped promoters, SINEs, and enhancers

Data S3. Gene Ontology of ERFS-associated genes

Data S4. ERFS-overlapped enhancers and their associated genes

Data S5. CNVs and SNVs identified in this study

Data S6. ERFS hotspots of hESCs

Data S7. ERFS hotspot-correlated genes

Data S8. Transcribed genes whose promoter contains ERFSs

Document S2. Article plus supplemental information
