# Supplementary material for: Early replication fragile sites are associated with cancer-related CNVs and SNVs in human embryonic stem cells
Source: Stem Cell Reports. 2026 Jun 18;21(7):102968. doi: 10.1016/j.stemcr.2026.102968 (PMC13385439; doi:10.1016/j.stemcr.2026.102968)
Supplement: Document S1. Figures S1–S5 [file mmc1.pdf]

Stem Cell Reports, Volume *21*

Supplemental Information

Early replication fragile sites are associated with cancer-related CNVs and SNVs in human embryonic stem cells

Yu-ping Dong, Menglin Qiu, Haoyu Tang, Wen Shi, Yi Lu, Fang Ji, Hongwei Liao, Songmin Ying, Ping Zheng, and Lin Wang

Supplemental Figures

A

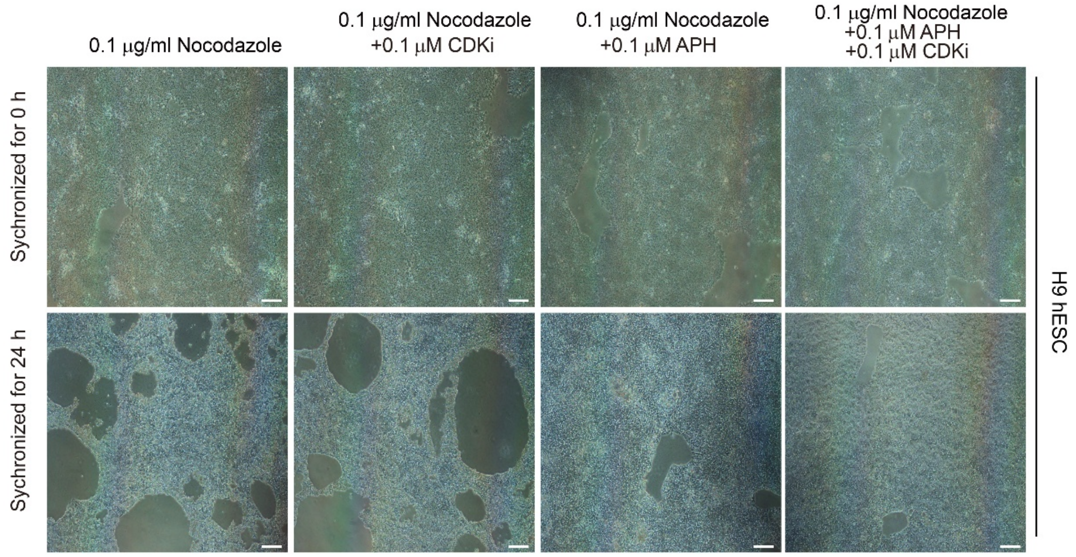

B

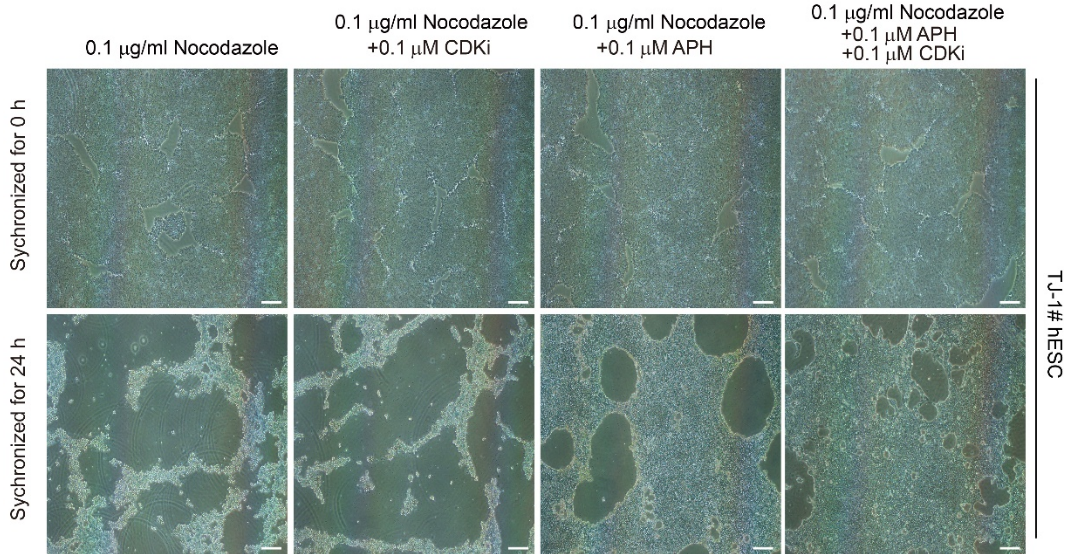

Figure S1. Assessment of G2/M phase synchronization efficiency in hESCs.

(A) H9 cells were treated with a combination of nocodazole plus low-dose aphidicolin (APH) and the CDK1 inhibitor (CDKi) RO-3306 for 24 h.(B) TJ-1# cells were treated with the same combined regimen of nocodazole, low-dose APH, and RO-3306 for 24 h. Treatment with nocodazole alone resulted in fewer surviving cells. Scale bar, 50 μm. Experiments were repeated 3 times with similar results.

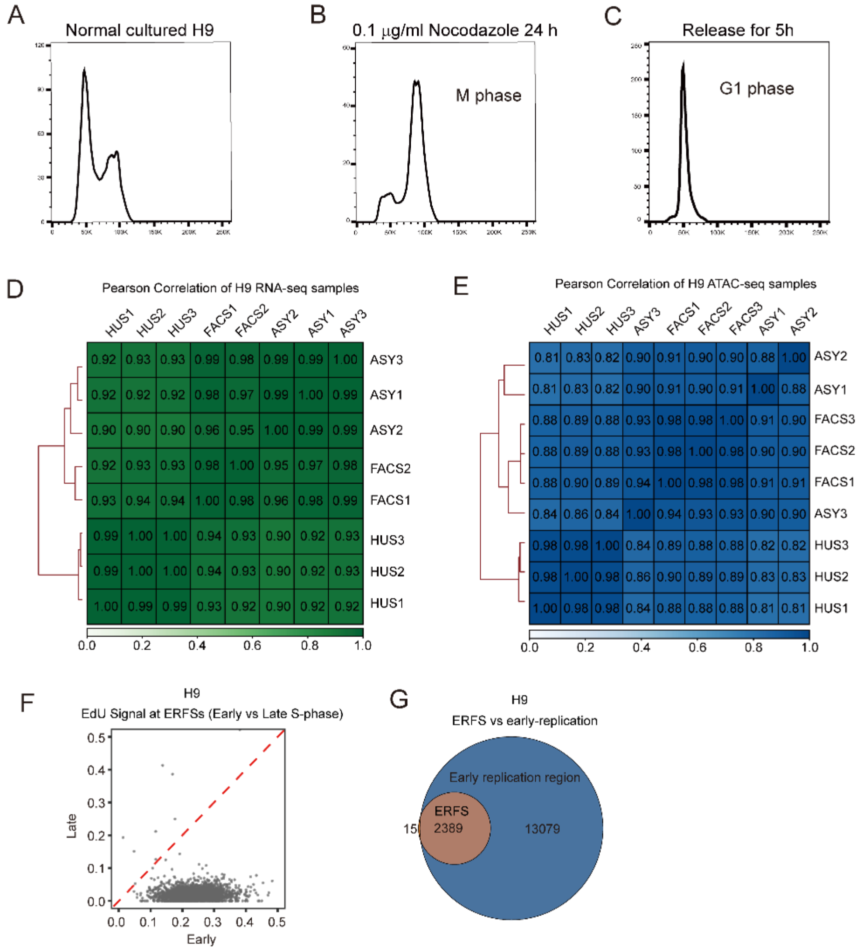

Figure S2. Quality control of cell synchronization in H9 cells.

(A) Cell cycle profile of asynchronously cultured H9 ESCs. (B) Validation of M phase synchronization by flow cytometry (FACS). (C) Validation of G1 phase synchronization by flow cytometry (FACS). (D) Pearson correlation analysis was conducted to compare the global transcriptomic profiles across synchronized early S-phase, FACS-sorted early S-phase, and asynchronous cells. (E) Pearson correlation analysis of global chromatin accessibility profiles across synchronized early S-phase, FACS-sorted early S-phase, and asynchronous cells. (F) Scatter plot of EdU-seq signal intensity at ERFSs in FACS-sorted early- and late-S phase fractions from asynchronous H9 cells. Each dot represents one ERFS. The red dashed line indicates y=x. (G) Overlap of drug-induced ERFSs with physiological early-replicating domains. Venn diagram showing the overlap between ERFSs identified by HU-induced synchronization (EH, n=2404) and early replicating regions defined by S50 estimator in asynchronous cells (AE, n=13094). The near-complete inclusion of EH within AE (2389/2404 EH loci overlap with AE) confirms that HU-

synchronized ERFS correspond to physiologically early replicating sites, validating the replication timing independence of the synchronization method. In (D-E), three biological replicates of HU-synchronized cells were labeled HUS1, HUS2, HUS3; three biological replicates of FACS-isolated early S-phase cells were labeled FACS1, FACS2, FACS3; and three biological replicates of asynchronous cells were labeled ASY1, ASY2, ASY3.

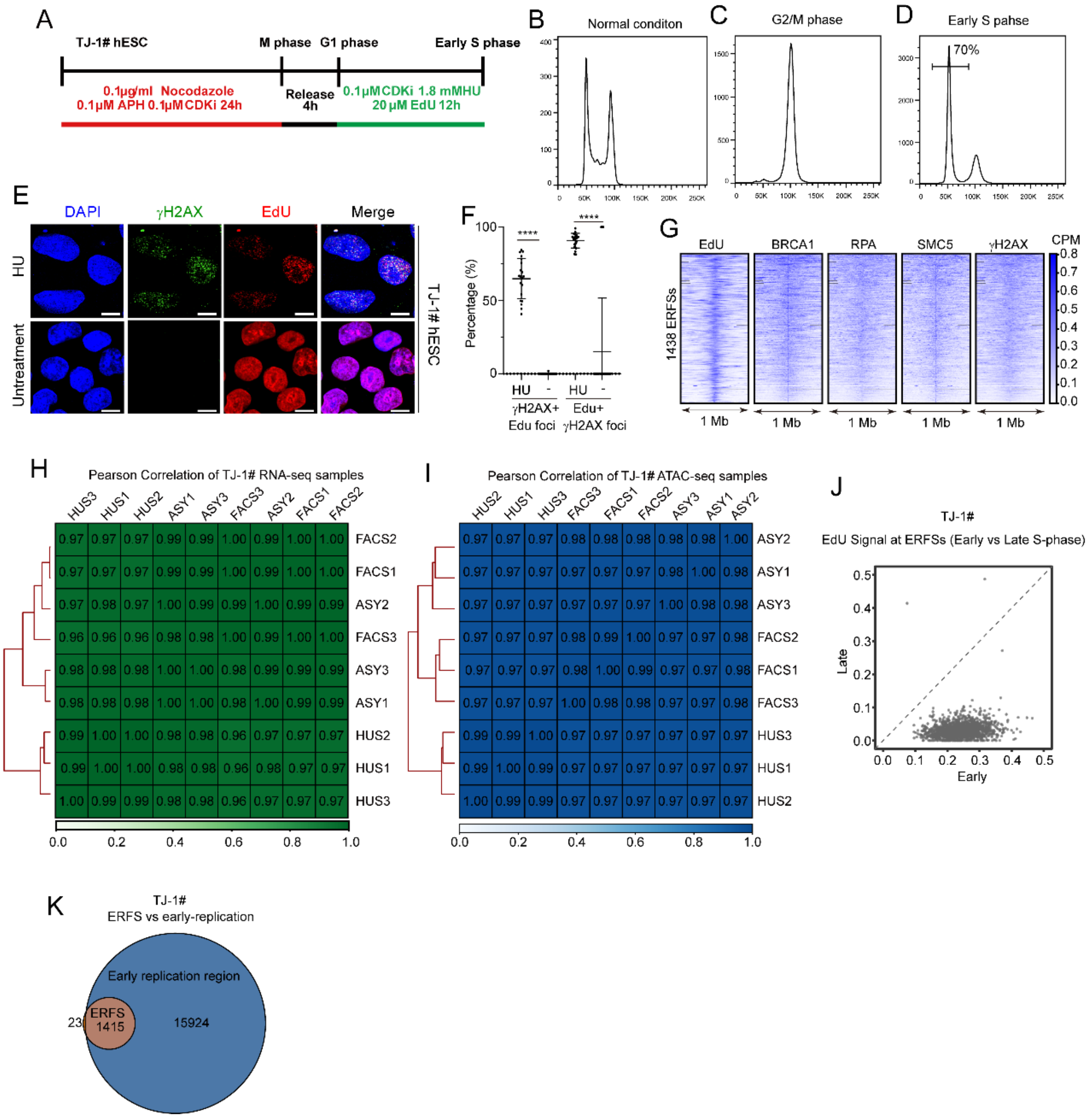

Figure S3. ERFSs induction and quality control of cell synchronization in TJ-1# cells.

(A) Schematic diagram of early S-phase synchronization for TJ-1# hESCs. (B) Cell cycle profile of asynchronously cultured TJ-1# hESCs. (C) Validation of G2/M phase synchronization by flow cytometry (FACS). (D) Validation of G1 phase synchronization by flow cytometry (FACS). (E) Images showing colocalization of EdU (red) with γH2AX protein (green) in nuclei after the TJ-1# cells were synchronized in early S-phase. Scale bar, 10 μm. (F) Quantification of the percentage of γH2AX foci that colocalized with EdU and the percentage of EdU foci that colocalized with γH2AX. A total of 20 individual cells were

quantified per group. Data are shown as mean ± SD. Mann–Whitney U test, ****$P < 0.0001$. (G) Heatmap of ERFSs from TJ-1# cells distribution on chromatin. ERFSs are identified by colocalization of EdU, BRCA1, RPA, SMC5, and γH2AX. (H) Pearson correlation analysis of global transcriptional profiles across synchronized early S-phase, FACS-sorted early S-phase, and asynchronous TJ-1# cells. (I) Pearson correlation analysis of global chromatin accessibility profiles across synchronized early S-phase, FACS-sorted early S-phase, and asynchronous TJ-1# cells. (J) Scatter plot of EdU-seq signal intensity at ERFSs in FACS-sorted early- and late-S phase fractions from asynchronous TJ-1# cells. Each dot represents one ERFS. The red dashed line indicates y=x. (K) Overlap of HU-induced ERFSs with physiological early-replicating domains in TJ-1# cells. Venn diagram showing the overlap between ERFSs identified by HU-induced synchronization (EH, n=1438) and early replicating regions defined by S50 estimator in asynchronous cells (AE, n=15924). The near-complete inclusion of EH within AE (1415/1438 EH loci overlap with AE) confirms that HU-synchronized ERFS correspond to physiologically early replicating sites, validating the replication timing independence of the synchronization method. In (H-I), three biological replicates of HU-synchronized cells were labeled HUS1, HUS2, HUS3; three biological replicates of FACS-isolated early S-phase were labeled FACS1, FACS2, FACS3; and three biological replicates of asynchronous cells were labeled ASY1, ASY2, ASY3. At least 20 fields were randomly analyzed in (E).

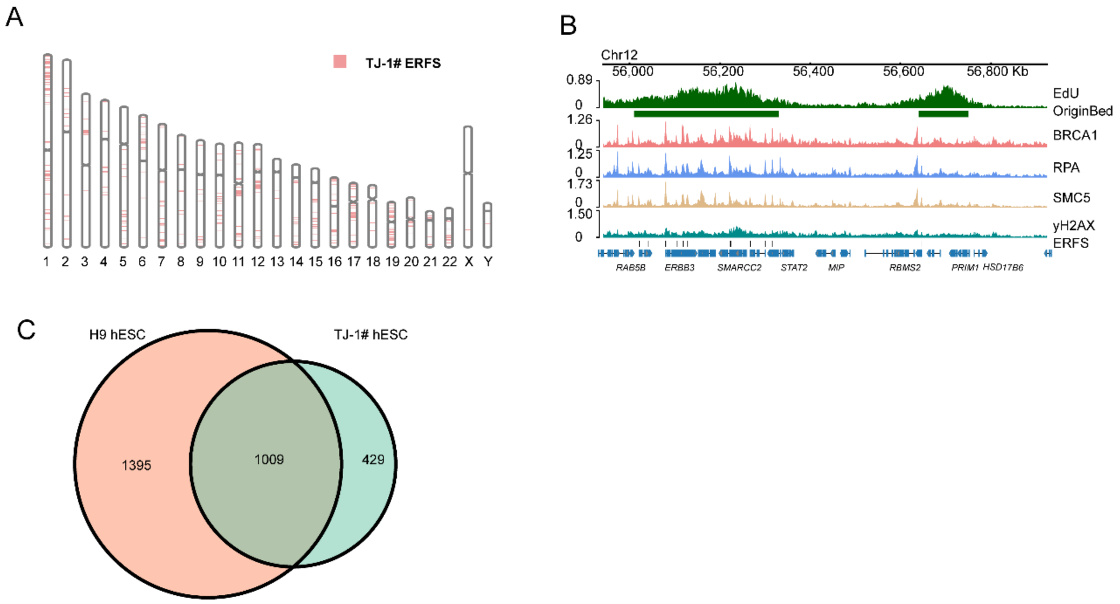

Figure S4. Genomic distribution of ERFSs in TJ-1# hESCs.

(A) Chromosomal distribution of ERFSs in TJ-1# hESCs. (B) Representative ERFS regions in TJ-1# hESCs. Genome browser tracks (from top to bottom) show EdU incorporation and occupancy of BRCA1, RPA, SMC5, and γH2AX on chromosome 12. The y-axis represents signal intensity in Counts Per Million (CPM). Selected genes were listed. (C) Venn diagram showing the overlap of ERFSs identified in H9 and TJ-1# hESCs.

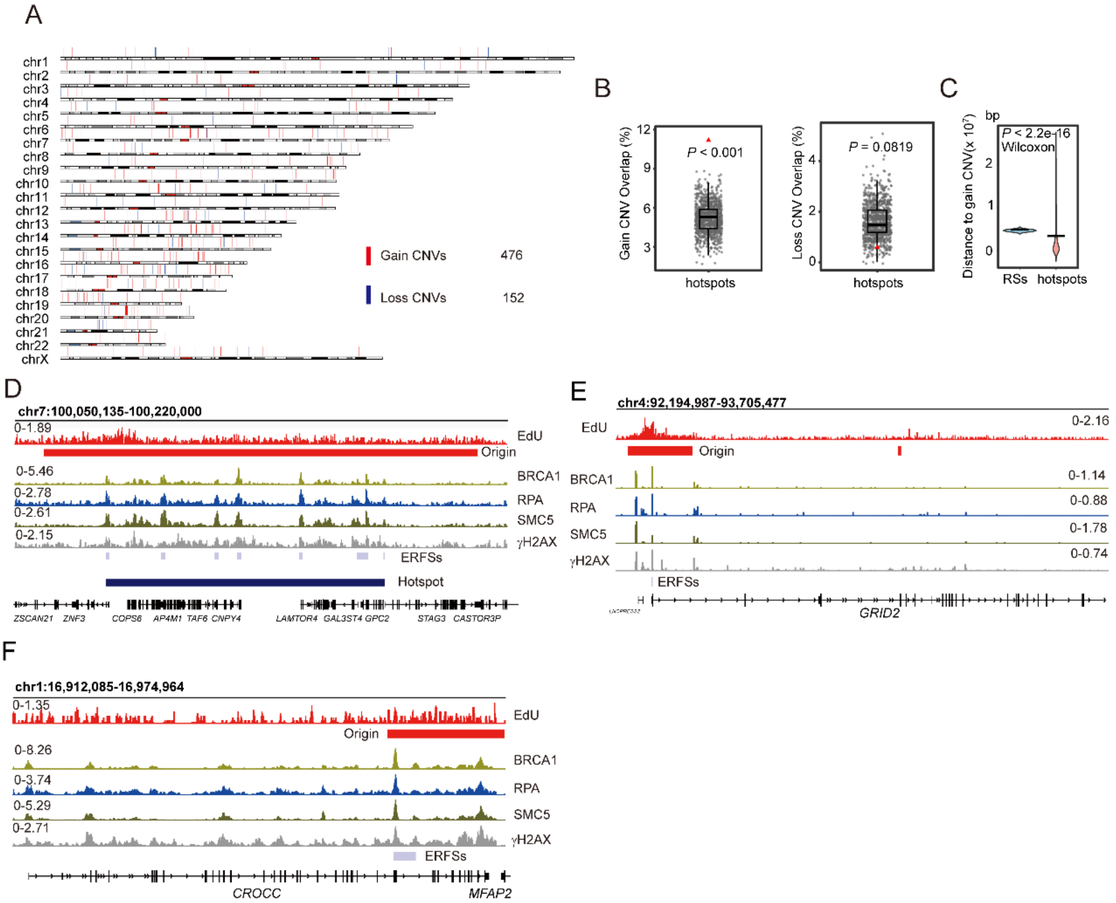

Figure S5. CNV distribution and ERFS-related CNV loci not identified in this study.

(A) Chromosome view of the distribution of CNVs identified in this study. (B) Percentage overlap of ERFS hotspots with CNV gain regions (left) and CNV loss regions (right) relative to randomly generated control regions. Box plots show the distribution of overlap values from random controls, with individual iterations shown as dots and observed hotspot values indicated by red triangles. ERFS hotspots show increased overlap with CNV gain regions (P<0.001), whereas overlap with CNV loss regions is observed at P=0.0819. P values were derived from permutation testing. (C) Violin plots show the distribution of linear distances from random control sites (RSs) and ERFS hotspots to the nearest CNV gain regions. Black horizontal bars indicate the mean distance for each group. (D-F) Genomic view of the representative ERFS-related CNV loci not identified in this study, including *AP4M1*, *GRID2*, and *CROCC*.
